# Supplementary material for: Content-rich biological network constructed by mining PubMed abstracts
Source: BMC Bioinformatics. 2004 Oct 8;5:147. doi: 10.1186/1471-2105-5-147 (PMC528731; doi:10.1186/1471-2105-5-147)
Supplement: Additional File 2 — The original results of the above study (non-essential files are deleted to keep the file size under the limit set by BMC bioinformatics). [file 1471-2105-5-147-S2.bz2 › chilibotAdditionalFile2/dip30/158ID7685215E560/html/FOS_TBP.html]

 


 **FOS** and **TBP** 
  
Found 30 abstracts in PubMed, retrieved 30.  
 

 What does Google say? 
 PDF only 
| .edu only 

---

**Interactive relationship** (e.g. stimulation, inhibition, etc)

**Neutral relationship**- High affinity binding to  **TBP**  requires c  **Fos**  activation modules which cooperate to activate transcription.  Ref: 8065335 Mol Cell Biol, 1994
- The topical application of 50 microg cm2 BaP  [ **TBP** ]  to EpiDerm resulted in the accumulation of BaP  [ **TBP** ]  DNA adducts and c  **fos**  and p53 proteins as evidenced by immunohistochemical localization.  Ref: 9920731 Biochem Biophys Res Commun, 1999
- Using co immunoprecipitation and protein protein association assays, we show that both c  **Fos**  and c Jun bind specifically and stably to  **TBP** .  Ref: 7685215 Gene Expr, 1993
- Mutational analysis demonstrates that both the basic region and leucine zipper domains of c  **Fos**  and c Jun are necessary and sufficient for stable association with  **TBP** .  Ref: 7685215 Gene Expr, 1993
- The bZIP domains of  **Fos**  and Jun mediate a physical association with the TATA box binding protein  [ **TBP** ] .  Ref: 7685215 Gene Expr, 1993
- We conclude that the E1A dependent activation of c  **fos**  transcription is mediated through an effect on a TATA binding protein  [ **TBP** ]  that has specificity for the TATAA sequence.  Ref: 2137244 Proc Natl Acad Sci U S A, 1990
- To examine protein protein interactions with cellular proteins at the visna virus promoter, we used an in vitro protein affinity chromatography assay and electrophoretic mobility shift assay, in addition to an in vivo two hybrid assay, to show that the visna virus Tat protein specifically interacts with the cellular transcription factors  **Fos**  and Jun and the basal transcription factor  **TBP**  TATA binding protein  [ **TBP** ] .  Ref: 9847304 J Virol, 1999
- Since  **TBP**  binding is not exhibited by Fra 1,  **TBP**  mediated activation may be one characteristic that discriminates the function of  **Fos**  related proteins.  Ref: 8065335 Mol Cell Biol, 1994
- We propose that c  **Fos**  and c Jun proteins function as transcriptional activators, in part by recruiting  **TBP**  to form complexes to initiate RNA synthesis.  Ref: 7685215 Gene Expr, 1993
- Collectively, these results suggest that c  **Fos**  is able to activate via two distinct mechanisms, only one of which requires contact with  **TBP** .  Ref: 8065335 Mol Cell Biol, 1994
- A 51 residue region from the conserved C terminal region of  **TBP** , previously shown to be the binding site for the viral activator protein E1A, interacts with c  **Fos**  and c Jun proteins.  Ref: 7685215 Gene Expr, 1993
- Exposure of quiescent SMCs to 30 microM BaP  [ **TBP** ]  inhibited the ability of serum to induce c  **fos**  mRNA expression and decreased AP 1 binding to a 12 O tetradecanoyl phorbol 13 acetate responsive element.  Ref: 7726552 Arch Biochem Biophys, 1995
- This inhibition squelching of c  **Fos**  activity is relieved by the presence of excess  **TBP** , indicating that  **TBP**  is a direct functional target of c  **Fos** .  Ref: 8065335 Mol Cell Biol, 1994

**Non-interactive relationship** (e.g. studied together, co-existance, homology, etc.)

- Strikingly, the first modification observed is methylation of histone H3 lysine 4, which correlates with the binding of the SET9 methyltransferase and the assembly of a complex consisting of c Jun, c  **Fos** , TATA binding protein  [ **TBP** ] , and RNA polymerase II.  Ref: 12588998 Mol Cell Biol, 2003
- BaP  [ **TBP** ]  MeoA cells acquired the greatest proliferative enhancement and exhibited unregulated c jun and c  **fos**  gene expression under growth arrested and serum stimulated conditions.  Ref: 7487084 Arch Biochem Biophys, 1995
- We find that the ability to stabilize the  **TBP**  TFIIA complex on the hsp70 and c  **fos**  TATA elements, and not on the SV40 early and E2A TATA elements, correlates with the ability or inability to activate promoters containing these TATA elements.  Ref: 9632777 Mol Cell Biol, 1998
- c  **Fos**  induced activation of a TATA box containing promoter involves direct contact with TATA box binding protein  [ **TBP** ] .  Ref: 8065335 Mol Cell Biol, 1994
- However, the levels of p53 and ras proteins and the levels of p53, c myc and c  **fos**  transcripts in the HOK 16B BaP  [ **TBP** ]  T cells were not different from those in the HOK 16B BaP  [ **TBP** ]  cells.  Ref: 7784058 Oncogene, 1995
- Mutation of either Tyr248, Tyr357, or Tyr462 to phenylalanine reduced transcription from a c  **fos**  promoter relative to wild type BAP  [ **TBP** ]  TFII I in transfected COS 7 cells, consistent with the interpretation that phosphorylation at these sites contributes to transcriptional activation.  Ref: 11373296 J Biol Chem, 2001
- Here we show that c  **Fos** , but not a related protein, Fra 1, can bind the TATA box binding protein  [ **TBP** ]   **TBP**  both in vitro and in vivo.  Ref: 8065335 Mol Cell Biol, 1994
- These includes nuclear factor,  **FOS**  related antigen 1, cytoplasmic dynein light chain, replication factor C, nucleoside diphosphate kinase B, UV excision repair protein, insulin like growth factor receptor II, transcription initiation factor TFIID  [ **TBP** ]  subunit, growth factor receptor bound protein 2, UV excision repair protein, glutathione peroxidase, Y box binding protein 1 and heat shock protein 86.  Ref: 10946852 Life Sci, 2000
- The ability of c  **Fos**  and c Jun proteins to interact directly with the TATA box binding protein  [ **TBP** ]   **TBP** , the general transcription factor required for initiating the assembly of transcription complexes, was investigated.  Ref: 7685215 Gene Expr, 1993
- c  **fos**  and c Ha ras gene expression was enhanced in liver, but not kidney, slices by BaP  [ **TBP** ] . c jun mRNA levels were decreased in liver and kidney slices, although the effect was earlier 0.5 h in liver slices compared to kidney slices.  Ref: 9852999 Toxicol Appl Pharmacol, 1998
- The association between Tat,  **Fos** , and Jun would position Tat proximal to the viral TATA box, where the visna virus Tat activation domain could contact  **TBP**  to activate viral transcription.  Ref: 9847304 J Virol, 1999
- It is the regulatory subunit of the DNA dependent protein kinase that phosphorylates many proteins, including SV 40 large T antigen, p53, RNA polymerase II, RP A, topoisomerases, hsp90, and many transcription factors such as c Jun, c  **Fos** , oct 1, sp 1, c Myc, TFIID  [ **TBP** ] , and many more.  Ref: 10755664 Crit Rev Biochem Mol Biol, 2000
